# The Ecology of Microbial Communities Associated with *Macrocystis pyrifera*

**DOI:** 10.1371/journal.pone.0067480

**Published:** 2013-06-19

**Authors:** Vanessa K. Michelou, J. Gregory Caporaso, Rob Knight, Stephen R. Palumbi

**Affiliations:** 1 Department of Biology, Hopkins Marine Station, Stanford University, Pacific Grove, California, United States of America; 2 Department of Biological Sciences, Northern Arizona University, Flagstaff, Arizona, United States of America; 3 Argonne National Laboratory, Institute for Genomics and Systems Biology, Argonne, Illinois, United States of America; 4 Department of Chemistry and Biochemistry, University of Colorado, Boulder, Colorado, United States of America; 5 Howard Hughes Medical Institute, Boulder, Colorado, United States of America; University of New South Wales, Australia

## Abstract

Kelp forests are characterized by high biodiversity and productivity, and the cycling of kelp-produced carbon is a vital process in this ecosystem. Although bacteria are assumed to play a major role in kelp forest carbon cycling, knowledge of the composition and diversity of these bacterial communities is lacking. Bacterial communities on the surface of *Macrocystis pyrifera* and adjacent seawater were sampled at the Hopkins Marine Station in Monterey Bay, CA, and further studied using 454-tag pyrosequencing of 16S RNA genes. Our results suggest that *M. pyrifera*-dominated kelp forests harbor distinct microbial communities that vary temporally. The distribution of sequence tags assigned to Gammaproteobacteria, Alphaproteobacteria and Bacteriodetes differed between the surface of the kelp and the surrounding water. Several abundant Rhodobacteraceae, uncultivated Gammaproteobacteria and Bacteriodetes-associated tags displayed considerable temporal variation, often with similar trends in the seawater and the surface of the kelp. Bacterial community structure and membership correlated with the kelp surface serving as host, and varied over time. Several kelp-specific taxa were highly similar to other bacteria known to either prevent the colonization of eukaryotic larvae or exhibit antibacterial activities. Some of these kelp-specific bacterial associations might play an important role for *M. pyrifera*. This study provides the first assessment of the diversity and phylogenetic profile of the bacterial communities associated with *M. pyrifera*.

## Introduction

Most aquatic organisms, particularly primary producers such as macroalgae, interact with their environment through their surface [1]. Bacteria-alga interactions vary from symbiotic to parasitic relationships that mainly depend on environmental parameters, such as the availability of inorganic nutrients and organic matter. In eutrophic coastal marine systems rapid bacterial biofilm growth on available surfaces takes place. This bacterial colonization is especially rapid if the surface is a potential source of nutrients, such as polysaccharides in exudates of kelp that may serve as source of carbon for heterotrophic bacteria living on the kelp surface [2], [3]. Kelp exudates may shape bacterial community composition [4], and create communities that are kelp-specific rather than randomly assembled from the surrounding seawater [5]–[7].

The giant kelps are important foundation species that can perform essential roles in coastal kelp forest ecosystems, and represent an important natural resource, providing shelter and a growth substrate for many species of fish, invertebrates, other seaweeds and even microbial biofilms on their surface [8]. Kelp forests along the central coast of California are of tremendous importance for coastal biodiversity, productivity, and the human economy in the region, and the bacteria associated with their surfaces are believed to be important in carbon and nitrogen turnover in kelp forest food webs [9], [10]. Recently, cultivation-independent methods have shed light on kelp-surface-associated microorganisms from kelps of the genera *Laminaria* and *Saccharina* [11], [12]. Bacterial communities associated with different structural features of the kelp differ in composition, and these communities differ seasonally and geographically [11]. However, the ecology of microbial communities on the predominant kelp found along the western California coast, *Macrocystis pyrifera*, has not yet been investigated. For example, it is not known which taxa are present on *M. pyrifera* surfaces or how they compare with the surrounding environment.

This study reports an in-depth description of the diversity and phylogenetic association of the microbial communities associated with *Macrocystis pyrifera*. We compared microbial communities, using 454 sequencing of the bacterial 16S rRNA gene, from the surface of kelp and the surrounding water in Monterey Bay in March, April and May, to investigate the composition and temporal dynamics of kelp-associated bacterial communities. Our results revealed a distinct epiphytic microbial community associated with these macroalgae, which provides a foundation for understanding the microbial ecology of kelp forests.

## Methods

### Sample collection and DNA extraction

Samples of *Macrocystis pyrifera* were taken from the site of the Marine Life Observatory in Monterey Bay (Hopkins Marine Station) in March, April and May 2010. Duplicate kelp blades (lamina) were sampled from two different individual fronds adjacent to each other, from one-meter depth by scuba diving. Kelp blades were, removed with a knife and transferred into sterile plastic bags. In close proximity to the kelp (within 1 m distance), two water samples of one liter each were collected separately before kelp sampling, in sterile Duran bottles. Additional duplicate one-liter water samples were also collected outside the edge of the kelp forest approximately 1 km away, following the procedure described above. All water samples were kept at in situ water temperature (13.1°C in March, 14.4°C in April and 11.4°C in May) until immediate processing upon arrival in the laboratory, within one hour after sampling. Additional kelp and water samples were collected at ten meters depth in May. Only water samples from inside and outside the kelp forest perimeter were analyzed during April 2010 as the kelp samples from this time point failed to amplify.

Kelp blades were washed three times in sterile seawater to remove loosely associated bacterial cells. The lower part of the lamina (meristematic region) was sampled. Areas that were heavily epiphytized were avoided. Bacterial DNA was extracted from the surface of the kelp blades as described by [13], which leaves the algal host intact and extracts total DNA from the microbial community on the entire surface. Briefly, a surface of approximately 50 cm^2^ from each meristematic region of every individual kelp blade was placed into 100 ml of calcium- and magnesium-free artificial seawater (CMFSW) containing 0.45 M NaCl, 10 mM KCl, 7 mM Na_2_SO_4_, and 0.5 mM NaHCO3 and supplemented with 10 mM EDTA and 1 ml filter-sterilized rapid multienzyme cleaner (OSM low-foaming multienzyme detergent; Fisher cat. #15-336-507) in 100 ml Erlenmeyer flasks. Samples were then incubated for 2 hours at room temperature and 80 rpm and then vortexed for 2 minutes. Kelp material was removed and the remaining liquid was centrifuged at 300×g for 15 min to remove any remaining algal material. The supernatant was filtered onto a 0.2 µm pore-sized Durapore filters and DNA was extracted from the filters by using a Cetyltrimethylammonium Bromide (CTAB) protocol involving two chloroform extractions and a high-salt isopropanol precipitation [14]. The extracted DNA was not pooled, and each sample represents the DNA from the surface community of a single kelp blade. Ten random segments of kelp (5 from before and 5 from after enzyme treatment) were stained with 5 µM SYTO9 nucleic acid stain (Invitrogen, Carlsberg, CA) and examined by light microscopy to assess that the kelp tissues were intact without any visible lesions (data not shown).

Between 1.3 and 1.8 liters of seawater were vacuum filtered through 0.22-μm-pore-size Durapore membrane filters (Millipore) at the laboratory, with two replicates per sampling location. Filters were then preserved in a CTAB buffer and stored at −80°C until DNA extractions were performed. DNA was extracted from the filters by using the same CTAB protocol mentioned above involving two chloroform extractions and a high-salt isopropanol [14]. DNA was eluted with 30 µl water and samples were diluted accordingly to a final concentration of 20 ng/μl. DNA samples were quantified using a Nanodrop spectrophotometer (Nyxor Biotech, Paris, France).

### 16S rRNA gene amplicon generation and 454 sequencing

Multiplexed bacterial tag-encoded FLX amplicon pyrosequencing (bTEFAP) was performed using the Titanium platform (Roche Applied Science, Indianapolis, IN) as previously described [15] in a commercial facility (Research and Testing Laboratories, Lubbock, TX). Briefly, a single step PCR using the primers that span the variable regions V1–V3 of the 16S gene, 28F 5′GAGTTTGATCNTGGCTCAG and 519r 5′GTNTTACNGCGGCKGCTG), was used to amplify the 16S rRNA genes as well as to add adaptor sequences and sample-specific 8-mer oligonucleotide tags (barcodes) to the amplicons. A total of 30 PCR cycles were run using a mixture of HotStart and HotStar high fidelity taq polymerases (Qiagen).

### Sequence analysis and statistical analyses

Sequence analysis was performed using the Quantitative Insights into Microbial Ecology (QIIME) pipeline [16] (version 1.2.0-dev, svn revision 1755) using default parameters unless otherwise noted. Sequences were first screened for quality using the following parameters: minimum quality score of 25, minimum sequence length of 200 bp, maximum length of 1000 bp, and no ambiguous bases in the entire sequence or mismatches in the primer sequence. Any sequences not meeting these parameters were excluded from downstream analyses. Sequences were then sorted by barcode into their respective samples and the barcode and primer sequences were removed. The sequences were denoised using the QIIME denoiser [17] and operational taxonomic unites (OTUs) were clustered de novo from the denoised sequences using uclust [18] at 97% identity. A representative sequence for each OTU was chosen as the centroid of each cluster, and these representative sequences were aligned using PyNAST [16]. A phylogenetic tree was constructed using the FastTree program [19] for use in phylogenetic diversity calculations. Taxonomy was assigned using BLAST against the Silva database (prefiltered at 97% identity). Chimeras were removed from the reference set on the basis of identification as chimeric via ChimeraSlayer [20]. Organelle sequences were excluded from the downstream analysis by filtering out all of the sequences whose taxonomy assignment contained the text “chloroplast” or “mitochondria”.

Weighted and unweighted UniFrac distances were computed between all samples after subsampling all samples to an even depth of 510 sequences per sample to control for differing depths of sequencing across the samples (the minimum, median and maximum sequences per sample, prior to the even sampling, were 179, 16155, and 21698, respectively). Principal Coordinates Analysis (PCoA) was applied to visualize the differences between the sample types. To test whether microbial community differences between sample types (water versus kelp) were significantly greater than differences within sample type (water versus water and kelp versus kelp) we performed a Monte Carlo simulation based on 1000 iterations of shuffling sample labels. All beta diversity results were confirmed at a sampling depth of 5482 sequences per sample, which allowed inclusion of several samples that were initially dropped because their sequence coverage fell below the even sampling depth (data not shown). Alpha diversity was computed using the full data set at a depth of 1031 sequences per sample. The sequence data has been deposited in the European Molecular Biology Laboratory (EMBL) Nucleotide Archive with accession number ERP002019, and can also be found in the QIIME Database under the study id 820 (http://www.microbio.me/qiime/).

## Results

### Diversity of microbial communities

After denoising, 276,908 reads were used for the subsequent analyses. The sample libraries ranged from 37,330 to 3,199 reads. The reads were assigned to 4,080 operational taxonomic units (OTUs) at 97% sequence identity level. The kelp surface libraries had the highest abundance of chloroplast and mitochondrial sequence contamination, ranging from 12–92% of all the sequences. The total number of bacterial sequences per library before and after the chloroplast and mitochondria removal is listed in Table 1. The very low diversity found on the kelp surface in March was due to the dominance of one OTU belonging to a chloroplast (OTU 858). This OTU formed nearly 84% of the sequences in the kelp surface sample taken in March.

**Table 1:** Summary of the sequencing results.

Date	Sample	Chloroplast	%	Mitochondria	%	Bacteria	%	97% OTUs	Chao1
March	Water in	4239	17.8	923	3.9	18676	78.3	232	600
		2502	14.5	696	4.0	14009	81.4	228	498
	Water out	5376	19.0	1150	4.1	21698	76.9	193	515
		5321	22.2	1100	4.6	17538	73.2	204	423
	Kelp	13321	90.0	1300	8.8	179	1.2	38	51
		31695	84.9	5125	13.7	510	1.4	62	137
April	Water in	2654	11.1	1141	4.8	20117	84.1	204	325
		2828	11.6	1288	5.3	20289	83.1	199	383
	Water out	209	3.5	276	4.6	5482	91.9	154	213
		944	5.2	861	4.7	16382	90.1	207	308
May	Water in 0m	157582	92.6	460	0.3	12148	7.1	238	553
		5020	21.1	727	3.1	18056	75.9	256	912
	Water out 0m	2065	10.3	680	3.4	17292	86.3	243	710
		2223	11.9	589	3.1	15928	85.0	263	783
	Kelp 0m	12550	63.0	1394	7.0	5990	30.0	98	154
		4518	76.0	295	5.0	1130	19.0	144	174
May	Water in 10m	4013	19.5	661	3.2	15855	77.2	243	565
		2420	10.8	791	3.5	19129	85.6	265	778
	Water out 10m	2617	12.7	538	2.6	17483	84.7	240	650
		2655	12.9	459	2.2	17462	84.9	230	644
	Kelp 10m	5090	33.7	489	7.4	1031	15.6	94	125
		2228	69.6	447	14.0	524	16.4	45	49

Abbreviations: OTU, operational taxonomic units

*Water In* samples were taken in close proximity to the kelp, inside the kelp forest; *Water Out* samples were sampled outside the kelp forest perimeter. The number of reads and the percentage of total sequences are displayed, categorized as being bacterial or organelle derived. OTUs at 97% identity and Chao1 OTU richness were estimated after sub sampling all samples to an even depth of 1031 sequences per sample to control for differing depths of sequencing across the samples (the minimum, median and maximum sequences per sample, prior to the even sampling, were 179, 16155, and 21698, respectively), and after removing organelle-derived sequences. Sampled depth for all samples and dates was 1 meter, unless otherwise noted.

### Bacteral community composition on Macrocystis pyrifera and in kelp forest waters

**Figure 1:**
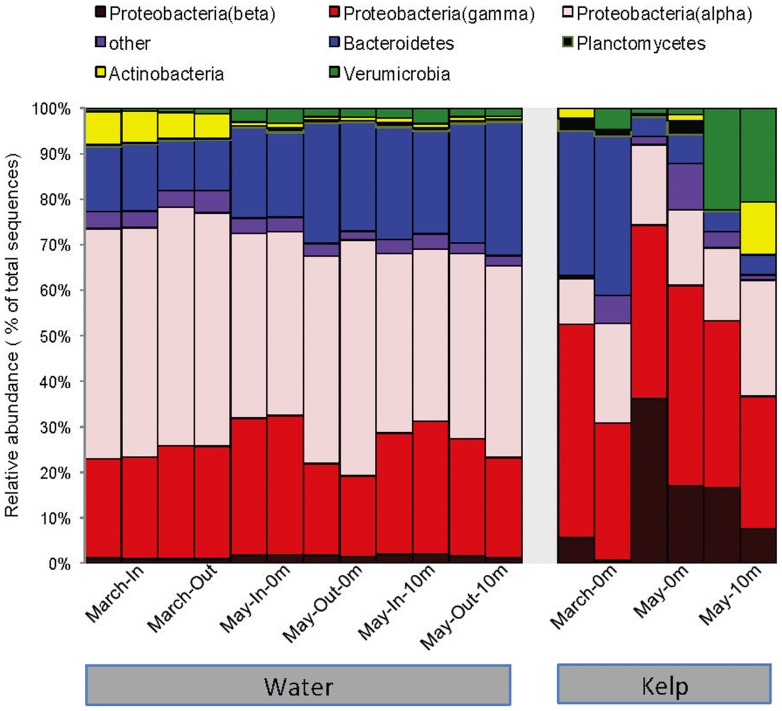
Kelp surface and seawater bacterial communities at the level of phyla. “Kelp” refers to the kelp surface samples; “Water-In” refers to the seawater samples adjacent to the kelp sampled; “Water-Out” refers to the seawater sampled outside the perimeter of the kelp forest.

At the phylum level, kelp surfaces and seawater samples generally had similar community composition (Fig. 1). The seawater samples inside and outside the kelp forest were predominantly composed of Alphaproteobacteria, Gammaproteobacteria, and Bacteroidetes. We found 20 bacterial phyla in at least one sample, with the most abundant groups within the Proteobacteria (38.1% of the sequences), Bacteroidetes (22.5%), Actinobacteria (9.0%), Verrucomicrobia (9.0%) and Planctomycetes (5.3%). The dominant phylotypes of the kelp surface environment, both abundant and widespread in kelp and/or water samples, were members of the Gamma-, Beta-, and Alphaproteobacteria within the Proteobacteria, and the Bacteroidetes (Table 2). The relative abundances of these dominant OTUs were highly variable between March and April/May samples and between the kelp surface and overlying seawater (Table 2). For instance OTU 1941 represents an uncultured member of the Pseudomonadales that made up more than 30% of the kelp surface sequences in May but was not detected in any of the seawater samples from any of the times sampled. Similar patterns were observed with other dominant phylotypes including OTU 741 (Burkholderiales order), OTU 6384 (Rhodobacteriales order) and OTU 55 (SAR11 clade) (Tables 2 and 3).

**Table 2:** Most dominant OTUs in all sites sampled for this study and their average relative abundances (as percentages of all sample 16S rRNA gene sequences recovered).

Water March	Water May	Kelp March	Kelp May	OTU#	Nearest neighbor	% ID	Class	Order
NA	NA	2	31.3	741	*Aquabacterium*	91	Betaproteobacteria	Burkholderiales
NA	NA	2.4	17.3	3619	*Verrucomicrobiaceae*	100	Verrucomicrobiae	Verrucomicrobiales
NA	NA	NA	31.9	1941	*Pseudomonas*	100	Gammaproteobacteria	Pseudomonadales
NA	NA	10.3	0.7	3882	*Flavobacteriaceae*	100	Flavobacteria	Flavobacteriales
NA	NA	4.6	1.4	3991	*Stenotrophomonas*	99	Gammaproteobacteria	Xanthomonadales
2.7	1.6	0.1	0.0	5417	*Ulvibacte*	100	Flavobacteria	Flavobacteriales
5.2	28.3	0.6	0.6	6384	*Thalassobacter*	83	Alphaproteobacteria	Rhodobacterales
5.0	12.6	0.1	0.1	4492	*Oleiphilaceae*	87	Gammaproteobacteria	Oceanospirillales
32.1	9.2	NA	NA	55	*Pelagibacter*	100	Alphaproteobacteria	SAR11

Abbreviations: OTU, operational taxonomic units; Phylogenetic classification was determined by BLAST against the Silva database.

OTUs were considered dominant if they were both highly abundant and occurred frequently in kelp samples. “NA” indicates that the OTU was not included within the 10 most dominant for that sample.

**Table 3:** OTUs at 97% similarity, which were found in all kelp surface samples, presented as their contribution to the whole community (% of the total bacterial sequences).

Taxon	Classification	March	May-0m	May-10m
Alphaproteobacteria	Sphingomonadaceae	12.7%	2.1%	1.8%
Alphaproteobacteria	Rhizobiales	12.7%	7.0%	3.1%
Betaproteobacteria	Burkholderiales	3.1%	26.2%	2.6%
Gammaproteobacteria	Pseudomonadales	8.1%	30.4%	38.4%
Gammaproteobacteria	Group2	8.3%	1.1%	0.5%
Gammaproteobacteria	Chromatiales	8.2%	2.0%	4.6%
Bacteroidetes	Flavobacteriaceae	18.9%	3.1%	1.8%
	TOTAL	71.96%	71.86%	52.74%

Abbreviations: OTU, operational taxonomic units; Classification indicates the taxonomical affiliation of the OTU sequences, and the level of taxonomic classification chosen included at least 99% of all sequences for a particular OTU.

**Figure 2:**
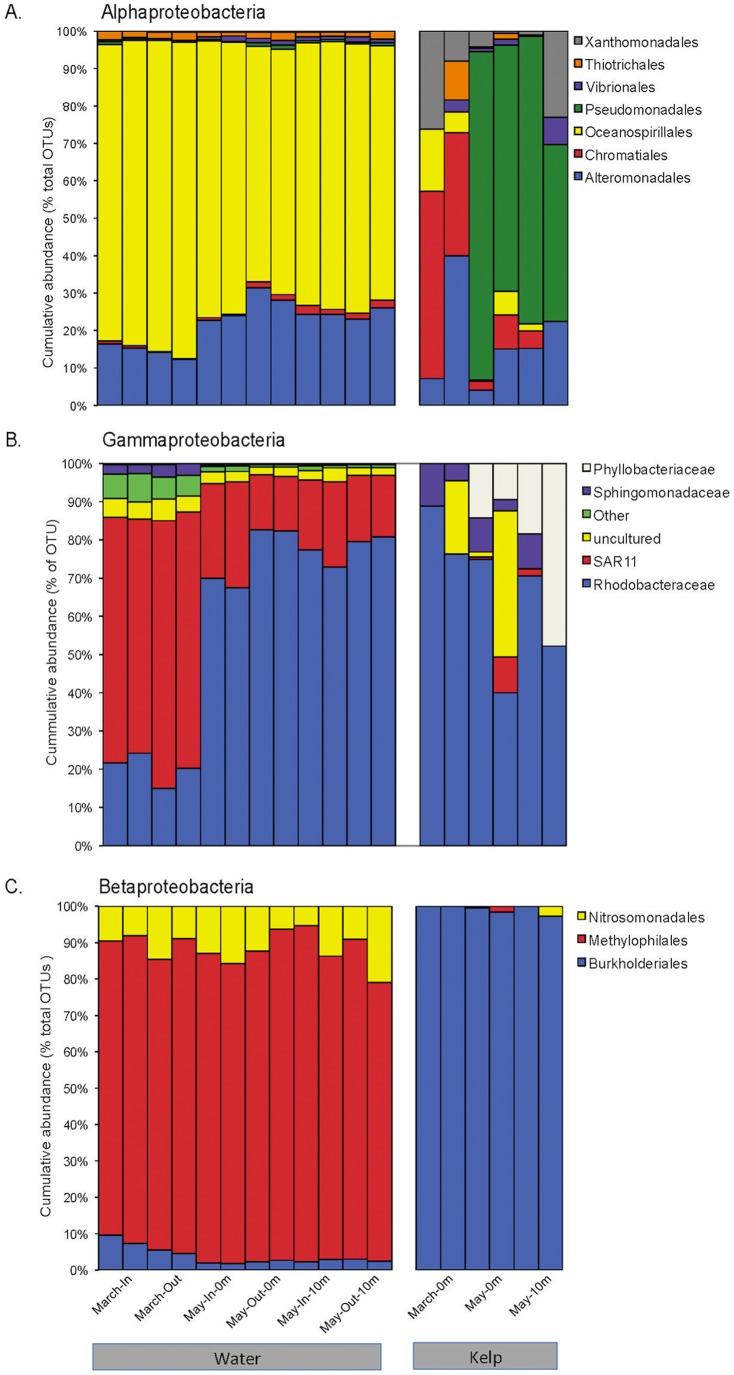
Bacterial distribution of the most abundant groups within the predominant classes of the Proteobacterial phylum. The *Alpha*- (A), *Gamma*- (B) and *Betaproteobacteria* (C) were consistently present in high abundances in all of the samples. A further evaluation within these classes showed differences in the profiles and abundance of the bacterial groups in seawater and on the kelp surface. X-axis sample designation is the same as Figure 1.

Considerable differences between the water column samples and the kelp surface were evident at finer levels of phylogenetic resolution. At the class level, the differences among the kelp and water samples were significant (one-tailed, two-sample t-test parametric p = 1.27×10^−50^, non-parametric p< 0.001). The seawater was dominated by Alphaproteobacteria (60% of total sequences), while the kelp surface was dominated by Gammaproteobacteria (55%). These differences among samples were also evident at the order level (one-tailed, two-sample t-test parametric p  = 1.27×10^−50^, non-parametric p< 0.001), as seen by the different composition of the main classes within the Proteobacteria (Alpha, Beta and Gamma) in the seawater and kelp surface samples (Fig. 2).

Within the Alphaproteobacteria class (Fig. 2A), SAR11 and Rhodobacteriales were particularly abundant in the water samples. Together, these two groups accounted for 85% to 95% of all of the sequences in the water with alternating prevalence of SAR 11 in March and Rhodobacteriales in May. The kelp samples were dominated by the Rhodobacteriales, constituting over 60% of all the bacterial sequences present (Fig. 2A) and to a lesser extent the Phyllobacteriales that were present only in May. In the seawater, 69% of the Gammaproteobacteria sequences were assigned to members of the order Oceanospiralles, which was not a major constituent of the kelp surface community (2% of all kelp sequences; Fig. 2B). Alteromonadales was the next most frequent order in the water samples, but was also rare in the kelp surface community. However, Pseudomonadales and Chromatiales dominated the communities on kelp samples, followed by a large number of uncultured Gammaproteobacteria sequences (8% of all kelp sequences). These two orders were rare in the seawater community (2% of all sequences in May). The third most abundant class of the Proteobacteria, Betaproteobacteria, was composed of the orders Methylophilales and Burkholderiales. The order Nitrosomonadales was also present, but it was much less abundant and only found in the seawater (Fig. 2C). Methylophiliales sequences dominated inshore and offshore seawater samples, while the kelp surface samples were almost entirely composed of OTUs from the Burkholderiales order, with very little variability between the surface and deep samples.

### Structure of kelp forest bacterial communities

**Figure 3:**
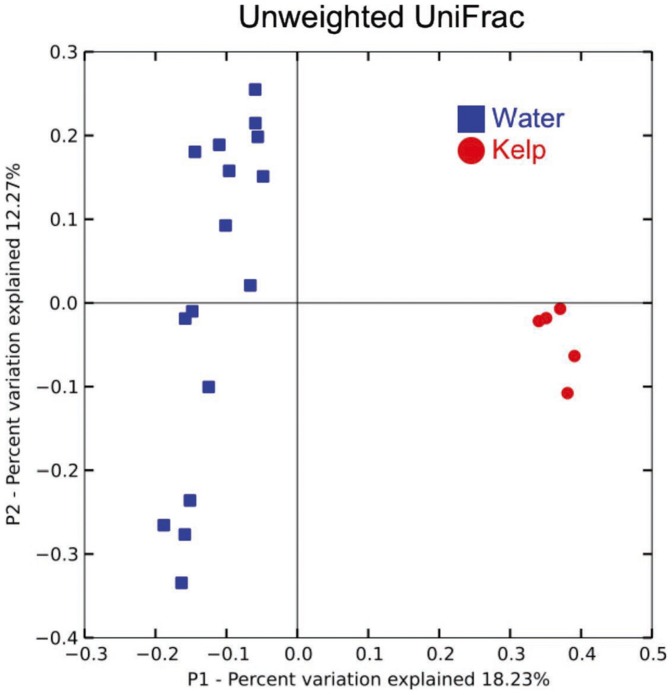
Unweighted UniFrac principal coordinates analysis (PCoA) plots computed at exactly 510 sequences/sample illustrate the relationship between sample type (blue  =  water, red  =  kelp) community similarities. Percentages of variance explained by each principal coordinate (P1 and P2) are shown on the x- and y-axes.

Principal Coordinates Analysis (PCoA) of unweighted UniFrac distances at exactly 510 sequence per sample show that the kelp surface and seawater communities harbour characteristic communities of Bacteria that differ from one another, evident by their independent clustering on the first principal coordinate axis (Fig. 3). This difference in community composition is statistically significant: the within-category distances (water-to-water and kelp-to-kelp) were significantly smaller than the between-category distances (water-to-kelp) when compared with both parametric and non-parametric t-tests (one-tailed, two-sample t-test parametric p-value = 1.27×10^−50^, Monte Carlo t-test p-value<0.001).

**Figure 4:**
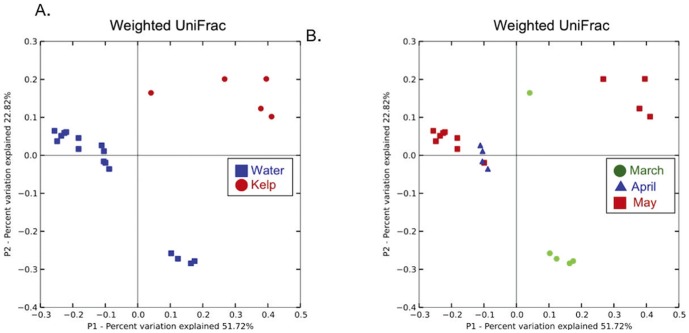
Weighed UniFrac principal coordinates analysis (PCoA) computed at exactly 510 sequences/sample illustrate the influence of sample type (Fig. 4A) and sample collection date (Fig. 4B). The coloring reflects the different dates on which the samples were taken (green  =  March, blue  =  April, red  =  May). “Kelp” refers to the kelp surface samples; “Water” refers to the seawater samples adjacent to the kelp sampled as well as those sampled outside the perimeter of the kelp forest. Percentages of variance explained by each principal coordinate (P1 and P2) are shown on the x- and y-axes.

Differences among bacterial communities were also assessed using weighted UniFrac, which takes into account the relative abundance of each OTU rather than presence/absence alone (Fig. 4). As with unweighed UniFrac, the between-sample-type distances (water-to-kelp) were significantly higher that the within-sample-type distances (Fig. 4A; one-tailed, two-sample t-test parametric p-value = 6.49×10^−14^, Monte Carlo p-value<0.001). A weighed UniFrac PCoA plot showing the samples colored by month of sampling (Fig. 4B) suggests that there may be seasonal patterns in the community composition of both kelp surfaces and surrounding sea water (which has previously been shown in sea water [21].

The data suggest that at least two factors affected community clustering: the sample type and the month of sampling. Along the PC1 axis (Fig. 4, 51.72% of the variance explained), the samples segregated by type. On PC2 (22.82% of the variance explained) we observe clustering of the water samples by sampling date: the March and April/May samples were the most different, although subtle differences occured between the April and May water samples. Shifts over time in the kelp samples were difficult to discern because the sampling occurred only in March and May, and in addition, one of the March samples had too few bacterial sequences (179) to be included in the analyses. Seasonal differences may thus occur in the kelp habitat, although a more detailed longitudinal study would be needed in order to demonstrate these differences conclusively.

## Discussion

### Seawater-associated communities

The community structure of bacterioplankton in the Monterey Bay region is well studied, with available data on the spatio-temporal structure of microbial populations and their response to episodic hydrodynamic events [22]–[25]. Our results from sequencing the 16S RNA gene from seawater samples inside and outside the kelp forest are in agreement with the published literature of coastal prokaryotic planktonic communities being dominated by Alphaproteobacteria, Gammaproteobacteria, Bacteroidetes, Actinobacteria and Betaproteobacteria [26], [27].

The most abundant OTUs from our seawater samples belong to known groups of bacteria characteristic of the surrounding area such as the Alphaproteobacteria SAR11, the Gammaproteobacteria from the *Roseobacter* clade and the Betaproteobacteria Methylophilales (Fig. 2A, B, C). One of the most abundant groups of Gammaproteobacteria we observed was the Oceanospirillales, which made up about 40% of the entire seawater community (Fig. 2A). The distribution of SAR11-related phylotypes in waters around the kelp forest with highest abundance in March, and much lower abundances in May (Table 2). We did not detect any seasonal changes in the predominance of the Betaproteobacteria or Gammaproteobacteria phylotypes in our seawater samples, and their distribution was constant throughout the study (Fig. 2B and C). Overall, the communities in both inshore and offshore water samples from the Monterey Bay kelp forest were identified as being typically marine-like.

### Dominant bacterial taxa associated with M. pyrifera surface

As in recent studies of surface-associated bacterial communities on sponges and macroalgae [7], the kelp and seawater communities were similar at the phylum level, with the both the water and kelp samples dominated by Proteobacteria (predominantly Alphaproteobacteria) and Bacteroidetes. The kelp surface libraries were characterized by sequences from the Rhodobacteraceae, Sphingomonadaceae (Alphaproteobacteria), Flavobacteraceae and Saprospiraceae (Bacteroidetes) families and included sequences from the Verrumicrobia and unclassified Gammaproteobacteria. These observations are in broad agreement with the relatively limited data regarding marine kelp-associated bacterial communities[11], [12], [28], [29].

The differences in the kelp and water community compositions may be due to the different nature of the physical environments harboring the two communities. The composition of the bacterial community can presumably be influenced by the chemistry of the kelp's surface, where metabolites and tissue composition can attract or repel certain bacteria resulting in communities comprised of bacterial groups adapted to kelp-surface lifestyles [30]. Kelp metabolites affect bacterial growth and attachment, and therefore, the composition of the bacterial community is influenced to some extent by the surface chemistry of the kelp. Metabolites and surface tissue composition may selectively attract or repel bacteria with patterns driven by the temporally variable nature of kelp exudates [4] and thereby shaping the microbial composition on its surface[31], [32].

It is known that kelp tissue concentrations of mannitol and laminarin increase during the winter and early spring, when growth rates are low, and are reduced (presumably to support growth) during the late spring and summer, when the onset of upwelling brings about high kelp growth rates [33]. Within the Alphaproteobacteria, Rhizobiales and/or Rhodobacterales were consistently found on kelp surfaces both in the March and April/May samples. OTUs closely related to sequences within the Rhizobiales comprised 10% and 9% of the March and May communities on the kelp surface, respectively. These bacterial groups are known for their antibacterial activity, suggesting that the kelp/bacteria symbiosis may be mutualistic with these groups assisting in defense of the kelp against potential pathogens. [34]

We expect bacteria that are capable of utilizing the dissolved carbon that is exuded from kelp cells as well as components of the extracellular mucus might be found on kelp surfaces. For example, the fucoidan-degrading activity of Verrucomicrobia may explain the abundance of this phylum on F. vesiculosus [35]. Similarly, Sphingomonodaceae, found in our study, have fucoidanolytic, alginolytic and polycyclic aromatic hydrocarbon (PAH)-degrading activities and may benefit from the kelp's surface components [36]–[39]. Bacteria belonging to the Bacteroidetes, Sphingobacteria and Actinobacteria have agarolytic and carrageenanolytic properties and may thus be attracted to cell-wall components of the kelp [40]. The March kelp surface harbored bacteria belonging to the Flavobacteriales, Sphingobacteriales, Xanthomonadales and Chromatiales. Bacteria belonging to the Bacteroidetes have been found to degrade complex polysaccharides, and Flavobacteriaceae strains have been isolated from rotting kelp, which may have similar characteristics to the kelp fronds from our March samples [41]. It is possible that the relatively high abundance of this group in such samples is linked to agarase-production, which could enable these bacteria to utilize agar from kelp fronds and benefit from exudates from old and damaged kelp tissue [37], [38], [40]. These kelp-surface associated bacteria may therefore represent an opportunistic collection of phylotypes that can colonize kelp tissue.

### Structure of kelp forest seawater bacterial communities

We found diverse sequence clustering patterns in the kelp and seawater samples examined, suggesting that bacterial community in the water column is distinct from the communities on the surface of the kelp. Although the seawater and kelp surface communities shared similarities at the phylum level, their bacterial communities were strikingly distinct at lower taxonomic levels. At the 97% OTU level, less than 2% of OTUs occurred in both the seawater and surface of the kelp.

We additionally observed temporal variation in the bacterial communities both on the surface of the kelp and in the water column. The relative abundances of the dominant bacteria from kelp surface samples changed between March and May (e.g., Gammaproteobacteria) (Table 2), but the abundances of other taxa (e.g. Alphaproteobacteria (Rhizobiales)) were largely unaffected by sampling time, indicating that the abundances of the latter groups may be influenced by other factors. Specific bacteria within Pseudomonadales and Burkholderiales were more abundant in May kelp samples, and as a result the entire surface community contained fewer members at this time. March kelp samples were more diverse, with 8 OTUs making up the majority of the surface community, whereas only three OTUs accounted for 81% of the kelp surface community in May (Table 3). We note that it is not possible to draw conclusions about seasonality of these communities since we sampled over only one three month period.

Seawater samples from inside and outside the kelp forest were similar in bacterial community composition during our study. Our sampling sites were close relative to the known dispersal distance for local waters (>4km) [42]. Since our sampling stations were less than 4 kilometers apart, it is not surprising that we found similar communities inside and outside the kelp forest. However, bacteria associated with the kelp surface exhibited a different pattern in community composition harboring different bacterial communities from those present in the water. The kelp surface may thus be acting as a highly specialized habitat for microbes distinct from that of the surrounding water column, selecting for growth of microbes that could be present in the water but perhaps at in very low numbers and that are able to thrive once they colonize the algal tissue.

In conclusion, we found several strong microbial community clustering patterns in the kelp and seawater samples examined, suggesting a bacterial community in the water column distinct from the communities on the surface of the kelp. Furthermore, bacteria associated with the kelp surface from the same locations sampled at different times exhibited different community compositions, suggesting temporal variation in kelp-associated microbial communities. Notwithstanding such dissimilarities, the communities in all the samples were typical of other marine environments. Future studies should address whether seasonal changes in environmental conditions, including temperature and nutrient load, affect community composition on the surface of kelp.
