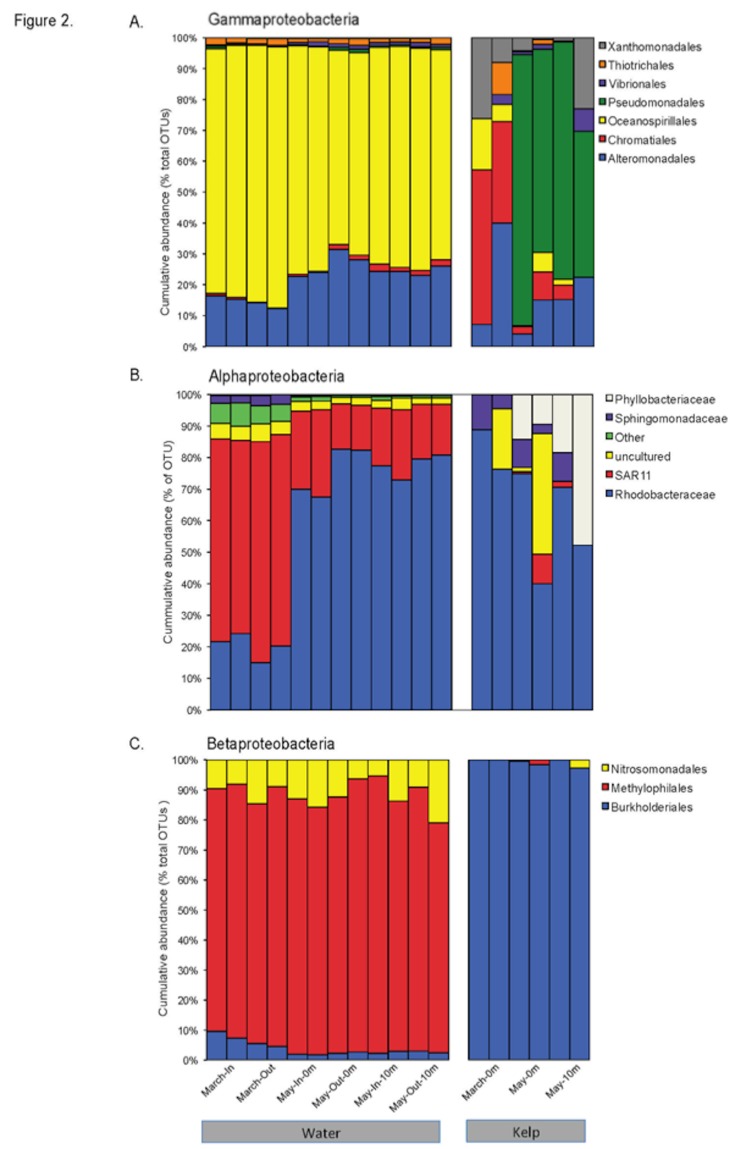# Correction: The Ecology of Microbial Communities Associated with *Macrocystis pyrifera*

**DOI:** 10.1371/annotation/48e29578-a073-42e7-bca4-2f96a5998374

**Published:** 2013-12-20

**Authors:** Vanessa K. Michelou, J. Gregory Caporaso, Rob Knight, Stephen R. Palumbi

The labels on panels A and B in Figure 2 were switched. Panel A should be labeled "Gammaproteobacteria" and panel B should be labeled "Alphaproteobacteria." Please see the corrected Figure 2 here: 

**Figure:**